# The potentiality of biostimulant (*Lawsonia inermis* L.) on some morpho-physiological, biochemical traits, productivity and grain quality of *Triticum aestivum* L.

**DOI:** 10.1186/s12870-023-04083-4

**Published:** 2023-02-14

**Authors:** Salwa A. Maksoud, Khaled I. Gad, Eman Y. M. Hamed

**Affiliations:** 1 Department of Botany and Microbiology, Faculty of Science, Cairo University, Giza, 12613 Egypt; 2 Wheat Department, Agricultural Research Center, Giza, Egypt

**Keywords:** Biostimulant, Wheat, Henna, Morphology, Biochemical traits, Productivity, Grain quality, Nutritional and bioactive compounds

## Abstract

**Background:**

In conformity with the international trend to substitute the artificial agro-chemicals by natural products to improve growth and productivity of crops, there is a necessity to focus on the environment sustainable and eco-friendly resources to increase crops productivity per unit area. One of these resources is the use of biostimulants. The aim of this study is to allow the vertical expansion of wheat crop by improving its growth and productivity per unit area as well as enhancing its grain quality using henna leaf extract as a biostimulant.

**Results:**

Field study was conducted to evaluate the potentiality of different doses of henna leaf extract (HLE) for improving the performance of wheat plants (*Triticum aestivum* L.) at three development stages. Results revealed that the response was dose dependent hence both 0.5 and 1.0 g/L doses significantly enhanced the growth of shoot and root systems, biochemical traits, yield and yield related components with being 1.0 g/L the most effective one. Furthermore, 1.0 g/L HLE markedly enhanced the quality of the yielded grains as revealed by increasing the content of soluble sugars (23%), starch (19%), gluten (50%), soluble proteins (37%), amylase activity (27%), total phenolics, flavonoids and tannins (67, 87 and 23%, respectively) as well as some elements including Ca (184%), Na and Fe (10%). Also, HPLC analysis of grains revealed that 1.0 g/L dose significantly increased the level of different phytohormones, soluble sugars and flavonoids (quercetin, resveratrol and catechin).

**Conclusion:**

Application of Henna (*Lawsonia inermis*) leaf extract at 1.0 g/L dose as a combination of seed priming and foliar spray can be recommended as a nonpolluting, inexpensive promising biostimulant, it can effectively enhance wheat growth, biochemical traits and productivity as well as improving the quality of the yielded grains.

## Background

Wheat (*Triticum aestivum* L.) is an important crop produced and consumed worldwide. It serves as the best cereal of choice, its grains supplies about 20% of the total dietary calories; rich in carbohydrates (78%), proteins (14.7%), lipids (2%), fibers, vitamins and minerals [1, 2]. Currently, wheat is considered as a second crop after rice in terms of dietary intake, with 68% of the wheat produced used for food, and the remains are consumed for feed and industrial biofuel [3]. Although wheat occupies the largest total harvested area (38.8%) among the cereals, its total productivity remains the lowest [4]. The rapidly growing population requires doubling the production of wheat crop by 2050 [5]. Egypt imports more than 50% of wheat requirements, thus we need new and rapid approaches to improve wheat productivity.

Biostimulant have essential role in improving the growth and productivity of plants through enhancing the efficiency of absorption and assimilation of nutrient [6]. They can be classified into five main groups based on the source of raw material: a) seaweeds and plant extracts containing bioactive substances) humic substances that mainly comprise humic and fulvic acids; c) hydrolyzed proteins and nitrogen containing compounds; d) micro-organisms that mainly include beneficial fungi, bacteria, and yeast, and e) inorganic compound with biostimulant action [6]. Biostimulants composition is not well defined; their mode of action is complicated due to the synergistic action of different compounds [6]. The use of biostimulants in crop management stills in early stages and need more investigation [7–9].

Biostimulants can enhance growth of various crops throughout several mechanisms based on improving physiological, biochemical and molecular aspects [10]. In general, biostimulant can act on primary metabolism by increasing photosynthetic pigments and sugars or accumulating secondary metabolites by activating specific metabolic pathways [6]. Furthermore, biostimulant can participate in ameliorating the level of several phytohormones [11–13] and proteins [14, 15]. Indeed, biostimulants can cause changes in many vital and structural processes thus increasing the yield and yield quality of crops [16, 17].

*Lawsonia inermis* L. (Henna) is a well-known medicinal and ornamental plant that has many biological and antimicrobial activities including antibacterial, antifungal, antioxidant, anti-inflammatory, anti-diabetic, anticancer and many other biological effects [18]. The role of HLE in improving plant growth is not well documented, the current study aimed to evaluate the efficiency of different doses of HLE in enhancing the growth and productivity of wheat plants as well as evaluating its influence on the quality of the resulted grains.

## Material and methods

### Preparation of biostimulant (henna leaf extract, HLE)

Henna leaf extractwas prepared by soaking (10 g) of dry leaves of *Lawsonia inermis* in deionized water with continuous shaking for 3 days followed by centrifugation at 5000 g for 10 min. The pellet was re-extracted twice and the supernatants were pooled.

### The experimental design

The material used was cultivated variety of *Triticum aestivum* L. (cv. Giza 171), kindly provided by the agriculture research center (ARC). This study was conducted in the field crop research institute of ARC, Egypt, for two successive seasons (2019–2020 and 2020–2021).Seed priming was done by soaking wheat grains in water and different concentrations of HLE ( 0.5, 1.0 and 5.0 g / L) for 6 h. Three replicates were cultivated on straight parallel lines (28 g grain/line) in a randomized complete block design; each replicate consists of three rows with three meters in length and 30 cm apart. Experimental research and field studies on cultivated wheat plants, including the collection of plant material, comply with relevant institutional, national, and international guidelines and legislation; all methods were performed in accordance with relevant guidelines and regulation.

Foliar application of different concentrations of HLE was carried out at three developmental stages (tillering, elongation and grain filling) with time interval 30 days after sowing (DAS). Plant samples were detached after one week from each spray; grains were collected at maturity stage (140 DAS).Growth criteria of wheat plants including morphological parameters, yield and its related components were assessed.

### Biochemical analysis of wheat leaves or grains

Photosynthetic pigments were determined using spectrophotometric method of Fadeel [19]. Total soluble sugars (TSS) and insoluble sugars were extracted according to Maness [20] and, Upmeyer and Koller [21], respectively. The amount of sugars was estimated using anthrone method described by Hedge and Hofreiter [22]. HPLC of individual sugars was analyzed by Agilent 1260 infinity HPLC Series (Agilent, USA) [23]. Phytohormones extracted from leaves and grains of wheat were analyzed using HPLC according to the China National Food Safety Standard [24] and Agilent application note 5991–5506 EN [25]. Soluble proteins were extracted and determined according to Lowry method [26]. The gluten content of wheat milling grains was evaluated following the method of American Association for Clinical Chemistry (AACC 38–10) [27]. α-amylase was extracted and the activity was determined according to Makkar et al. [28]. Free and glycosylated phenolics were extracted according to Sauvesty et al., [29] and Stalikas [30], respectively. Total soluble phenolic content was estimated using the method of Lowe [31]. Total flavonoid was extracted according to Sauvesty et al., [29] and determined by AlCl_3_ colorimetric method [32]. HPLC analysis of individual phenolic and flavonoid compounds of wheat grains were carried out according to the protocol of Agilent Application Note, publication number 5991-3801EN [33]. Total tannins were extracted according Alagesaboopathiet al., [34] and estimated as described by Schander [35]. Total terpenes were extracted and determined following the method Ghorai et al. [36]. For elemental analysis advanced microwave digestion system was used for digestion of samples according to manufacturer's recommendations [37].

### Statistical analysis

The values were expressed as mean ± standard deviation. Differences between groups were assessed by one-way analysis of variance (ANOVA) using statistical package for the social sciences (SPSS) software for Windows, version 16. Combined analysis over the two growing seasons was performed according to Gomez and Gomez, [38]. The mean comparisons among treatments were determined by Duncan´s multiple range test at 5% level of probability. All data subjected to analysis of means and standard deviation using Microsoft excel program.

## Results and discussion

### Growth criteria of wheat plants

During this investigation a combination of seed priming and foliar spray of wheat with various levels of plant biostimulant (0, 0.5, 1.0 and 5.0 g/L HLE) were applied. Different morphological and growth attributes of shoot and root systems were estimated and presented in Tables 1, 2 and 3. By aging all the studied growth criteria of shoot and root systems were markedly increased to reach its highest levels at grain filling stage. For all stages of growth, treatment with (0.5 and 1.0 g/L) HLE significantly increased all parameters in a dose dependent manner, meanwhile the highest dose (5.0 g/L) exhibited insignificant variation.

**Table 1:** Influence of different concentrations of biostimulant (HLE) on shoot height, fresh and dry weights of wheat plants at different developmental stages

Stage	Treatment	Height (cm)	F.W(g)	D.W (g)
Tillering	Control	28.27 ± 1.23 a	7.41 ± 0.61 a	1.26 ± 0.13 a
	0.5 g/L	31.23 ± 1.1 b	9.38 ± 0.67 b	1.66 ± 0.13 b
	1.0 g/L	33.85 ± 0.71 c	11.69 ± 0.71 c	2.05 ± 0.13 c
	5.0 g/L	26.93 ± 1.06 a	6.99 ± 0.53 a	1.12 ± 0.10 a
Elongation	Control	76.52 ± 1.69 a	78.30 ± 6.31 a	14.22 ± 0.99 a
	0.5 g/L	83.63 ± 3.69 b	105.25 ± 5.99 b	17.72 ± 1.73 b
	1.0 g/L	90.62 ± 2.26 c	121.19 ± 9.19 c	20.52 ± 0.94 c
	5.0 g/L	73.30 ± 4.61 a	73.42 ± 3.75 a	13.73 ± 0.66 a
Grain Filling	Control	97.33 ± 1.76 a	121.27 ± 10.16 a	25.32 ± 2.24 a
	0.5 g/L	104.33 ± 3.06 b	142.58 ± 8.18 b	32.37 ± 1.32 b
	1.0 g/L	111.50 ± 3.61 c	155.14 ± 7.13 c	37.23 ± 2.83 c
	5.0 g/L	99.00 ± 5.20 ab	114.97 ± 5.62 a	24.63 ± 1.27 a

Values are means of three replicates ± SD, each is mean of five plants

Mean values followed by the same letters within each column are not significantly different at 0.05 level

**Table 2:** Influence of various concentrations of biostimulant (HLE) on number of tillers, leaves and leaf area of wheat plants at different developmental stages

Stage	Treatment	No. of tillers	No. of leaves	Leaf area (cm^2^)
Tillering	Control	7.17 ± 1.08 a	24.17 ± 1.76 a	14.93 ± 1.24 b
	0.5 g/L	8.67 ± 1.53 b	27.83 ± 3.52 b	18.79 ± 1.20 c
	1.0 g/L	10.67 ± 0.79 c	29.50 ± 2.76 b	22.99 ± 1.77 d
	5.0 g/L	6.33 ± 1.08 a	21.50 ± 1.62 a	12.17 ± 1.14 a
Elongation	Control	8.33 ± 0.87 a	44.67 ± 5.10 ab	33.82 ± 3.38 b
	0.5 g/L	10.83 ± 0.54 b	49.50 ± 4.82 b	44.10 ± 4.01 c
	1.0 g/L	12.9 ± 1.05 c	59.33 ± 5.04 c	54.40 ± 3.22 d
	5.0 g/L	8.17 ± 1.26 a	43.17 ± 1.55 a	28.70 ± 1.59 a
Grain Filling	Control	9.17 ± 0.79 a	47.50 ± 3.81 a	54.21 ± 3.16 a
	0.5 g/L	11.83 ± 0.79 b	55.00 ± 3.89 b	70.14 ± 4.28 b
	1.0 g/L	13.33 ± 0.30 c	70.50 ± 6.74 c	77.23 ± 3.96 c
	5.0 g/L	9.00 ± 0.58 a	45.33 ± 3.08 a	51.01 ± 4.54 a

Values are means of three replicates ± SD, each is mean of five plants

Mean values followed by the same letters within each column are not significantly different at 0.05 level

**Table 3:** Influence of different concentrations of the biostimulant (HLE) on the root system of wheat plants at different developmental stages.

Stage	Treatment	Root System		
		Length (cm)	FW (g)	DW (g)
Tillering	Control	9.91 ± 1 1.25 a	1.27 ± 0.16 a	0.35 ± 0.02 a
	0.5 g/L	11.93 ± 1.02 b	1.42 ± 0.14 b	0.44 ± 0.02 b
	1.0 g/L	13.07 ± 1.21 b	1.44 ± 0.12 b	0.47 ± 0.02 b
	5.0 g/L	9.52 ± 0.80 a	1.19 ± 0.09 a	0.34 ± 0.02 a
Elongation	Control	11.67 ± 0.98 a	5.66 ± 0.38 a	2.00 ± 0.42 a
	0.5 g/L	13.87 ± 1.13 b	7.8 ± 0.69 b	2.75 ± 0.21 b
	1.0 g/L	14.75 ± 0.83 b	8.33 ± 0.67 b	2.88 ± 0.24 b
	5.0 g/L	11.23 ± 0.38 a	5.58 ± 0.45 a	1.85 ± 0.11 a
Grain Filling	Control	13.27 ± 1.17 a	10.19 ± 0.95 ab	3.45 ± 0.35 a
	0.5 g/L	15.23 ± 1.33 b	11.40 ± 0.88 b	3.99 ± 0.33 b
	1.0 g/L	16.43 ± 0.70 b	12.13 ± 0.89 c	4.46 ± 0.20 b
	5.0 g/L	13.07 ± 0.82 a	9.60 ± 0.87 a	3.34 ± 0.30 a

Values are means of three replicates ± SD, each is mean of five plants

Mean values followed by the same letters within each column are not significantly different at 0.05 level

Little is known about the bio-stimulating effect of henna extracts on the growth of plants. In this context, Chandrasekaran et al., [39] reported that treatment of soybean seed with 10% henna leaf extract significantly increased shoot length. The study of Hanafy et al., [40] revealed a positive influence of HLE on height; fresh and dry weights; and leaf area of *Schefflera arboricola* plants.

Also, growth parameters of Lemon grass were enhanced substantially by application of 8 g/L henna leaf extract, yet higher dose (16 g/L) showed insignificant variations [41].

Several authors reported the stimulatory effect of some biostimulants on the growth parameters of various plant species such as fenugreek on wheat [42], garlic extract on eggplants [43], *Lemna minor* on maize [44], *Artimisia vulgaris* on potato [45], *Eucalyptus* on quinoa [46] and moringa on *Phaseolus vulgaris* [47] and geranium plants [48]. Ahmad and coworkers, [48] attributed the bio-stimulating potential of *Moringa oleifera* to the presence of high levels of proteins, essential amino acids and minerals in its leaves.

### Photosynthetic pigments

**Fig. 1:**
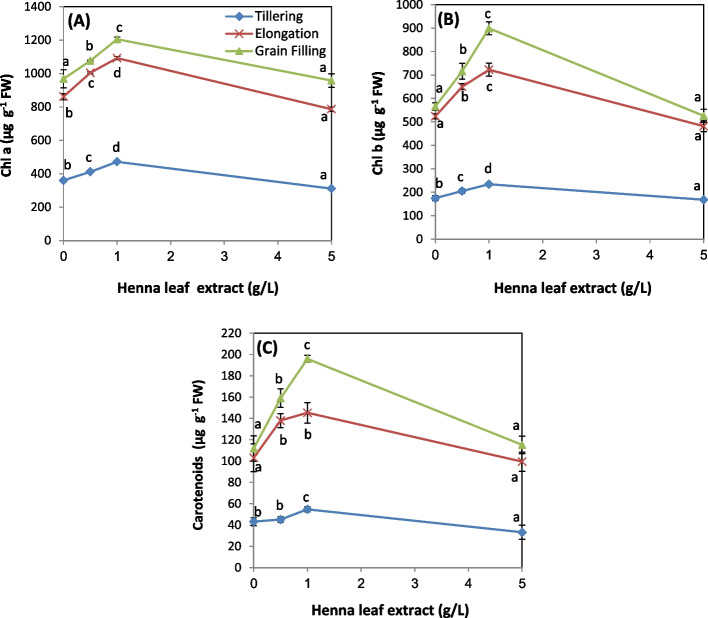
Influence of various levels of biostimulant (0.0, 0.5, 1.0 and 5.0 g/L, HLE) on the level of photosynthetic pigments of wheat plants at different developmental stages: **A**) Chl a, **B**) Chl b and **C**) carotenoids Values are means of three replicates ± SD. Values with different letters are significantly different from each other at *P* < 0.05 level according to Duncan´s Multiple Rang test

Figure 1A, B and C shows the changes in photosynthetic pigments of wheat leaves in response to treatment with different concentrations of HLE. By aging, any value of Chl a, Chl b and Car was elevated to record the highest level at grain filling stage. The two doses (0.5 and 1.0 g/L) significantly increased all photosynthetic pigment in a concentration dependent manner at any stage of growth. The optimum dose (1.0 g/L) increased the level of Chl a by (24, 27, 24%), Chl b by (34, 38, 59%) and Car (27, 41, 75%) for tillering, elongation and grain filling stages, respectively.

In the same line with our investigation, it was found that spraying lemon grass with henna extract (4 and 8 g/L) increased their Chlorophyll content about 11% [41]. Likewise, treating wheat, sunflower and lavender plants with fenugreek seed extract significantly increased their photosynthetic pigments [49]. Dawood and coworkers, [49] attributed the enhancements for the presence of important nutrient as Fe element which is an essential component of the Chlorophyll molecule. Likewise, application of moringa leaf extract (MLE) significantly increased the content of photosynthetic pigments in several plants such as *Hibiscus sabdariffa* [50], *Eruca sativa* [51], *Phaseolus vulgaris* [52], and *Cucurbita pepo* [53]. Spraying quinoa plants with different doses of garlic clove or Eucalyptus leaf extracts (5, 10 and 15%) caused significant elevation in the content of Chl a, Chl b and carotenoids in a concentration dependent manner [46]. Also, Chlorophyll content of maize plants was increased significantly in response to application of red grape skin and blueberry extracts [54]. Findura et al., [45] postulated that foliar treatment with *Artemisia vulgaris* exerts a positive effect on the content ofChl a, Chl b and Car of potato.

### Sugar content

**Fig. 2:**
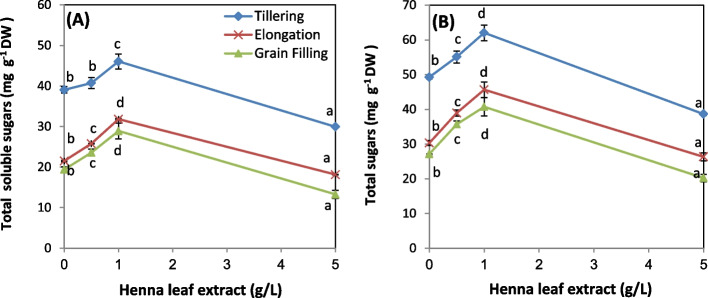
Influence of various levels of biostimulant (0.0, 0.5, 1.0 and 5.0 g/L, HLE) on the content of: **A** total soluble sugars and **B** total sugars of wheat plants at different developmental stages. Values are means of three replicates ± SD. Values with different letters are significantly different from each other at *P* < 0.05 level according to Duncan´s Multiple Rang test

Results of the current study (Fig. 2A, B) revealed that, the level of total soluble sugars (TSS) and total sugars (TS) exhibited the same pattern at all stages of growth; the early stage recorded the highest values. Treatment of wheat plants with (0.5 and 1.0 g/L) HLE gradually accumulated both types of sugars in all stages of growth with being 1.0 g/L dose the most effective one. Meanwhile, application of the highest dose reduced the content of TSS and TS to a level less than their corresponding controls.

In accordance with our data several biostimulants were found to increase the content of TSS and TS of different plants. Hanafy et al., [40] found that spraying *Schefflera arboricola* with HLE significantly increased total sugars about 18.5%. Similarly, total sugars were increased in response to treating wheat plants with fenugreek [49], rocket with moringa leaf and twig extracts [51] and quinoa plants with two extracts: garlic clove and Eucalyptus leaf extracts [46]. Likewise, spraying eggplants with garlic bulb extract increased their TSS by 112% compared to the control plants [42].

### Phtohormones content

**Fig. 3:**
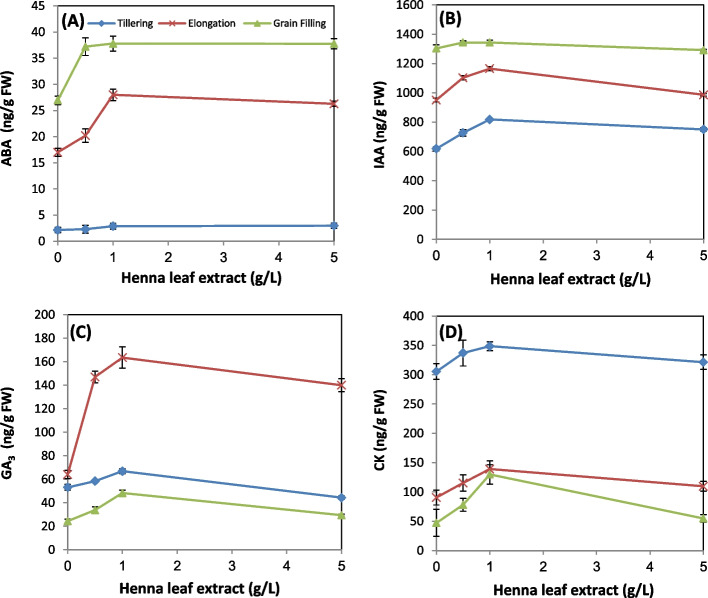
Influence of various levels of biostimulant (0.0, 0.5, 1.0 and 5.0 g/L, HLE) on phytohormones content of wheat leaves at different developmental stages. **A** Abscisic acid (ABA); **B** Indole acetic acid (IAA); **C** Gibberellic acid (GA_3_); **D** Cytokinin (CK). Values are means of three replicates ± SD. Values with different letters are significantly different from each other at *P* < 0.05 level according to Duncan´s Multiple Rang test

Data depicted in Fig. 3 illustrate phytohormones analyzed by HPLC. Concerning abscisic acid (ABA), results of the present investigation (Fig. 3A) revealed that application of any dose of HLE increased ABA level of wheat plants at elongation and grain filling stages, while at the first stage of growth, slight variation was recorded. In this context, a marked increase in the level of ABA was reported upon application of MLE to *Phaseolus vulgaris* plants [55]; while, the same treatment decreased ABA in the rocket plant [56]. Abscisic acid is a stress-responsive hormone that accumulated after perception of stress signals [57]. ABA can promote the synthesis of proline, antioxidant enzymes as well as the expression of various stress-responsive proteins like dehydrins and late embryogenesis abundant proteins [58]. Furthermore, ABA can modify the metabolism of primary lipids that participates in membrane-stress adaptive reorganization [59].

Data of Fig. 3B revealed that indole acetic acid(IAA) level of wheat leaves was markedly increased in response to application of HLE (0.5 and 1.0 g/L) at tillering and elongation stages, the most effective dose was 1.0 g/L with increment 32% and 23% for both stages, respectively. At grain filling stage, application of any dose of biostimulant resulted in slight variations in IAA level compared to the control. Inconsistent with our results, several researchers reported an increase in IAA level in response to biostimulant treatment. Application of chitosan (40 mg/L) to wheat plants increased IAA level about 63% [13]. Also, foliar application of MLE increased markedly IAA of rocket [51] and snap bean plants [60]. Similarly, application of Licorice root extract has been shown to increase IAA content in leaves of pear trees [11]. It was postulated that auxins control various physiological processes that regulate plant growth and development including cell elongation and apical dominance [61]. Indeed, Bouzroud and coworkers [62] reported that IAA plays an important role in root branching, differentiation of vascular tissue, fruit and flower development, and abiotic stresses.

Data of the present study (Fig. 3C) revealed that, gibberellic acid (GA_3_) level was elevated in response to application of (0.5 and 1.0 g/L) HLE at all developmental stages; the most effective dose was (1.0 g/L). The increments for 0.5 and 1.0 g/L doses were (10, 26%) at tillering, (130, 156%) at elongation and (39, 98%) at grain filling stage, respectively. The highest dose of biostimulant declined GA_3_ level (11%) at tillering stage and conversely increased its level at grain filling and elongation stages (20 and 118%, respectively).It is worth to mention that, the level of GA_3_ increased by aging to reach its maximum level at elongation stage then decreased again to the lowest level at grain filling stage. In accordance with our results, it was found that application of MLE to wheat, rocket and snap beans plants significantly elevated their levels of gibberellins compared to the untreated controls [14, 55, 63]. Also, the root extract of Licorice has been shown to elevate the content of gibberellins [9]. Elzaawely et al., [47] reported that the increase in the endogenous content of gibberellin especially (GA_7_) resulted in increasing leaf area, photosynthetic activity and yield. Gibberellic acid develops the growth criteria, photosynthetic pigments, nutritional values, rate of electron transport and energy trapping efficiency of PSII. Therefore, the crop yield of various wheat verities was improved due to better osmoregulation led to increased water flow by organic solutes [64, 65].

Data presented in Fig. 3D demonstrates that the highest level of cytokinin (CK) was recorded at the first stage of growth then decreased by laps of time to reach its lowest level at grain filling stage. The levels of CK were increased at all growth stages in response to sequenced application of any dose of HLE. The most effective dose was 1.0 g/L; the increments were (14, 54 and 175%) for the three successive stages. In the same line with our results it was reported that application of MLE was found to significantly accumulate CK in leaves of wheat and rocket plants compared to the unsprayed controls [14, 63, 66].

### Soluble protein content

**Fig. 4:**
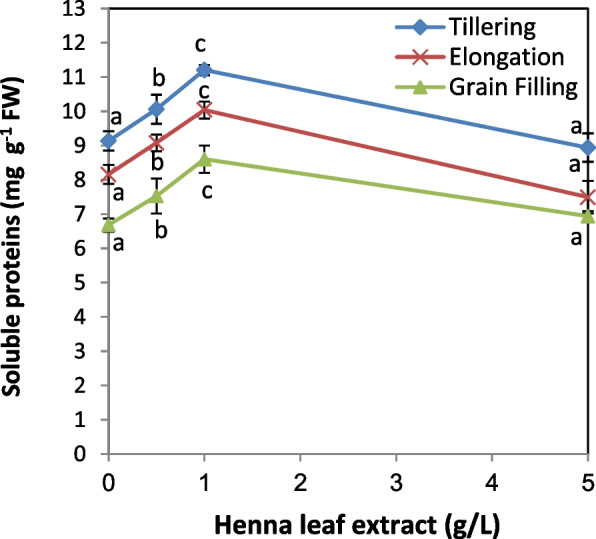
Influence of various levels of biostimulant (0.0, 0.5, 1.0 and 5.0 g/L, HLE) on soluble protein content of wheat plants at different developmental stages. Values are means of three replicates ± SD. Values with different letters are significantly different from each other at *P* < 0.05 level according to Duncan´s Multiple Rang test

Results of the present work (Fig. 4) indicated that treating wheat plants with HLE (0.5 and 1.0 g/L) significantly increased their soluble protein content in a dose dependent manner at the three developmental stages of growth. Application of the highest dose exhibited negligible changes at any developmental stage compared to the corresponding controls.

In agreement with our results, an elevation in the level of soluble proteins in response to treatment with various biostimulants have been observed in zucchini seedlings treated with cypress leaf extract [16], spinach and lettuce seedlings with microalgal extracts [67], maize plants with: red grape, hawthorn and blueberry extracts [54] and beans with seaweeds and yeast extracts [68]. Puglisi and coworkers, [67] attributed the enhancement of plant growth by biostimulant treatment to the elevation in the level of total soluble proteins.

Proteins play multiple functions in plant growth including synthesis of osmo-protectants, transporters and chaperones, proteases, detoxification of enzyme systems and act as a first line for direct protection from stress. In addition, regulatory proteins such as protein phosphatases and kinases, transcription factors, and activation of signaling molecules are essential in controlling the expression of signal transduction and stress-responsive genes [69].

### Yield and its components

**Fig. 5:**
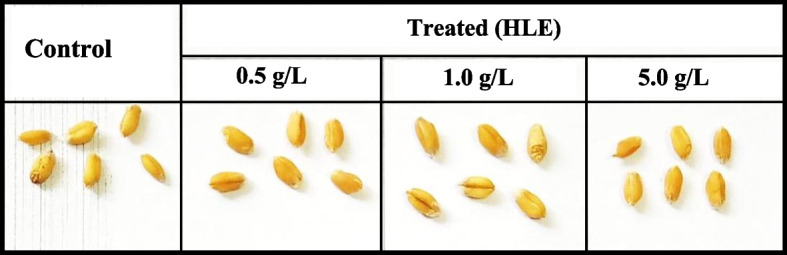
Grains yielded from control and biostimulant (HLE) treated plants

Table 4 illustrates the changes in wheat yield and its components in response to treatment with various levels of HLE. Data revealed that treatment with 0.5 and 1.0 g/L HLE significantly elevated the yield and its related parameters. The most effective dose was 1.0 g/L; it increased the grain yield about 32%. This increase was associated with elevation in various yield components including No. of spikes and grains/ plant (17, 21%, respectively) as well as weight of 1000 grain (Fig. 5) and weight of grains /plant (16 and 49%, respectively). The increase in grain yield and No. of tillers/plant (Table 2) explain the marked increase (35%) in the biological yield.

**Table 4:** Influence of different concentrations of biostimulant (HLE) on the yield of wheat plant and its components

Yield characters	Biostimulant concentration (HLE)			
	Control	0.5 g/L	1.0 g/L	5.0 g/L
Biological yield (Kg) / blot	8.41 ± 0.4 a	10.38 ± 0.74 b	11.30 ± 0.50 c	7.96 ± 0.24 a
Grain yield (Kg) / blot	2.11 ± 0.13 b	2.63 ± 0.10 c	2.86 ± 0.08 d	1.92 ± 0.13 a
No. of spikes / plant	12.33 ± 0.35 a	14.33 ± 0.27 b	15.63 ± 0.49 b	11.67 ± .52 a
No. of grains / spike	61.67 ± 2.53 a	62.67 ± 2.47 a	63.67 ± 3.03 a	61.0 ± 1.82 a
No. of grains / plant	761 ± 31.58 a	918 ± 21.14 c	984 ± 32.23 d	713 ± 28.93 a
Wt. of 1000 grain (g)	47.45 ± 1.54 b	51.63 ± 0.73 c	54.92 ± 1.91 d	44.83 ± 1.26 a
Wt. of grains (g) / plant	36.04 ± 1.42 a	47.91 ± 1.53 b	53.57 ± 2.97 c	31.99 ± 3.04 a

Values are means of three replicates ± SD, each is mean of five plants

Mean values followed by the same letters within each column are not significantly different at 0.05 level

According to various studies, biostimulants exhibited a positive effect on the yield of many plants [17, 70]. In agreement with our results, it was found that treatment of wheat plants with two plant extracts (*Sorghum helepense*; *Partheinum hysterophorus*) at a concentration (125 g/L) exhibited a significant increase in wheat biological and grain yields [71]. Also, Ali et al., [42] revealed that wheat plants treated with *Cuscuta reflexa* extract (CRE) recorded a significant increase in weight of 100 grain and grain yield, the maximum increment in grain yield was 14.8 and 12.32% upon treatments with 20 and 10% CRE, respectively. Nagwa and Iman, [53] reported that foliar spraying of wheat plants with different extracts (pomegranate, eucalyptus, cactus, garlic, and neem) significantly increased its yield and weight of 1000-grain.Additionally, Zida et al., [72] reported an increase in sorghum yield by treatment with *Eliptica alba* aqueous extract. Yakhin et al., [73] postulated that biostimulants treatment improved plant growth by stimulating germination, increasing plant metabolism, the absorption of nutrients from the soil and enhancing photosynthesis and thereby increasing plant productivity.

### Influence of 1.0 g/L HLE on the grain quality

#### Phytochemical screening

Date of Table 5 showed insignificant change in the percentage of germination between grains yielded from control and treated plants, however the average rate of coleoptiles and radicals growth was increased (43 and 28%, respectively). In this concern, it was found that priming *Pisum sativum* [74] and pepper [75] seeds with 3% MLE, exhibited an increase in the percentage of seed germination as well as the rate of root and shoot growth, compared to the control.

**Table 5:** The quality of wheat grains (germination characters and chemical constituents) yielded from control and biostimulant (1.0 g/L HLE) treated plants according to paired sample T- test

**Parameter**	**Grains**		**Change (%)**	**Paired sample** **T- test** at 95% Confidence
	**Control**	**HLE (1.0 g/L)**		
% of germination (three days)	93.33 ± 5.77	96.67 ± 5.77	3.6	**.423**
Coleoptile length (cm/three days)	0.93 ± 0.06	1.33 ± 0.06	43	**.020**
Radical length (cm/ three days)	1.43 ± 0.06	1.83 ± 0.06	28	**.020**
Amylase activity unit (µg maltose / g / min)	39.18 ± 3.21	49.81 ± 1.08	27	**.044**
Soluble proteins (mg/g)	9.33 ± 0.33	12.80 ± 0.21	37	**.002**
% of Fresh gluten /g	19.92 ± 0.14	30.55 ± 0.18	53	**.000**
% of Dry gluten /g	6.79 ± 0.06	10.03 ± 0.04	48	**.000**
Soluble sugars (mg/g)	49.33 ± 2.08	60.79 ± 1.71	23	**.006**
Total sugars (mg/g)	310.54 ± 18.16	371.46 ± 21.84	20	**.024**
Insoluble sugars (mg/g)	261.21 ± 17.73	310.67 ± 23.19	19	**.014**
Soluble phenolics (mg gallic acid /g)	1.59 ± 0.05	2.35 ± 0.03	48	**.002**
Total phenolics (mg/g)	6.48 ± 0.15	10.83 ± 0.09	67	**.001**
Flavonoids (µg/g)	78.48 ± 4.22	146.52 ± 6.66	87	**.008**
Tannins (µg/g)	380.80 ± 28.61	468.38 ± 22.31	23	**.009**
Terpenoids (mg/g)	13.93 ± 1.19	14.78 ± 0.49	6	**.404**

Amylase activity which has a role in seed germination was activated by 27% in the grains of biostimulant treated plants (Table 5). In this concern, it was found that priming wheat grains with CRE [42] and *Pisum sativum* seeds with 3% MLE [74] enhanced α-amylase activity. The increased level of α-amylase consequently accelerates the breakdown rate of the reserved materials into simple sugars; the resulted molecules are actively used as building blocks by the newly developing seedlings resulting in better germination and seedling establishment [76]. Furthermore, these molecules reduce the osmotic potential of grains resulting in higher water absorption [77].

In the current investigation, soluble proteins were accumulated in the obtained grains (37%) in response to biostimulant treatment (Table 5). It is well known that, protein content is one of the most important indicators of wheat grain quality hence; it determines the quality of the product end-use. Application of two seaweed extracts (*Kappaphycus* and *Gracilara* species) significantly increased the protein content of wheat grains by 15.6 and 13%, respectively [78]. Several authors reported an improvement of grain quality by increasing protein content in response to biostimulant application. For instance, treating rice with MLE [79], maize with 3% of four extracts (sorghum, moringa, maize and rice extracts) [80], and *Phaseolus vulgaris* plants with biostimulants containing seaweed or amino-acids [81].

**Fig. 6:**
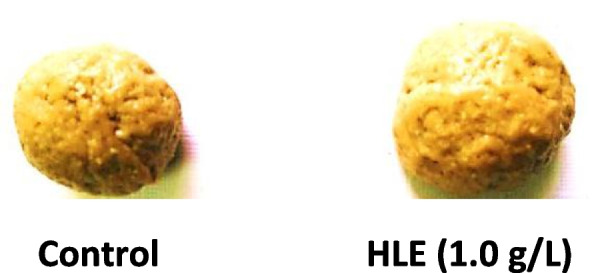
Gluten content of wheat grains obtained from control and HLE (1.0 g/L) treated plants

In the present work, fresh and dry weights of grain gluten (Table 5, Fig. 6) were markedly increased about 50% in response to biostimulant treatment compared to grains harvested from control plants. Gluten is a group of heterogeneous immune modulatory proteins rich in gliadin (confers extensibility) and glutenins (cause elasticity) complex with proline, glycine and glutamine [82]. The quality and quantity of protein, is important for dough properties and hence improve the bread-making quality of flour. There are a linear relationship between the high protein content and the quality of bread making [64].

Table 5 revealed that the level of soluble, insoluble and total sugars in wheat grains significantly elevated about 20%, upon treating plants with 1.0 g/L HLE. Khan et al., [79] reported an increase in the grain quality (amylose and amylopectin levels) of rice plants in response to MLE treatment. Likewise, starch content of maize grain was improved by application of 3% sorghum, moringa maize and rice extracts [80].

Data presented in Table 5 revealed that, the levels of soluble and total phenolic compounds, flavonoids as well as tannins were elevated in the grains by 48, 67, 87 and 23%, respectively in response to treatment; meanwhile, terpenes level showed a negligible increase. In consistent with our results, it was found that biostimulants containing seaweed or amino-acid extracts increased the quality of *Phaseolus vulgaris* seeds by increasing phenolics, and flavonoids [81].

### Element content

Table 6 illustrates the mineral composition of the grains yielded from control and treated plants. Marked increase in the level of Ca was recorded (184%) followed by Na and Fe (about 10%). Minor changes were recorded for Mg, Zn and S while, Mn level was decreased by 17%.

**Table 6:** Mineral composition of wheat grains of control and biostimulant treated plants (1.0 g/L HLE), expressed as ppm

Grains	Mg	Ca	Mn	Fe	Zn	S	Na	K
Control	0.127	0.019	20.315	27.952	24.802	0.113	0.0252	0.487
HLE (1.0 g/L)	0.131	0.054	16.893	30.672	25.437	0.118	0.0276	0.489
Change (%)	3.1	**184.2**	**-16.8**	**9.7**	2.6	4.4	**9.5**	0.4

Popko et al., [83] reported an increase in the mineral content of wheat grains such as Cu, Na, Ca and Mo upon treatment with two commercial biostimulants Amino-Prim and Amino-Hort; the increments were (35, 43%) for Na and (4.3, 7.9%) for Ca, respectively. Likewise, application of seaweed extract increased the micronutrient content such as Cu, Zn, Fe and Mn in rice grains [84]. The average mineral content of grains of different wheat species were (3.93, 42.8, 79.6 and 0.012 mg/kg) for Cu, Fe, Mn and Ca, respectively. No significant change in the mineral content of wheat grain was recorded upon treatment with organic fertilizers [85].

### HPLC analysis of some nutritional and bioactive compounds of wheat grains

#### Soluble sugars

HPLC analysis of individual sugars of the yielded wheat grains (Table 7) revealed the detection of four sugars, namely: sucrose, glucose, mannose and fructose. Application of biostimulant accumulated fructose, mannose and glucose (86.5, 62 and 10%, respectively) while, the content of sucrose was declined (20.5%); consequently the total content of the detected sugars was elevated by 22%. In this concern, Drobek et al., [7] recorded an increase in the level of glucose and sucrose in tomato fruits in response to treatment with *arbuscula rmycorrhiza* and *Pseudomonas* sp.

**Table 7:** HPLC analysis of soluble sugars of wheat grains of control and biostimulant (1.0 g/L HLE) treated plants, expressed as mg/g

Sugars	Control	HLE (1.0 g/L)	Change (%)
Sucrose	5.85	4.66	-20.45
Glucose	5.90	6.49	**9.98**
Mannose	0.93	1.51	**62.40**
Fructose	4.24	7.90	**86.47**
Total	16.92	20.56	**21.49**

### HPLC analysis of phytohormones in wheat grains

Table 8 shows the changes in the phytohormones content of grains yielded from control and treated plants. All the detected phytohormones was increased in response to biostimulant treatment, ABA and IAA gave the highest increments (38 and 33%, respectively), followed by GA_3_ (26%) then CK (9%). The total content of these hormones was increased by 33%.

**Table 8:** HPLC analysis of endogenous hormones of wheat grains yielded from control and biostimulant (1.0 g/L HLE) treated plants, expressed as ng/g

Hormones	C (0.0)	HLE (1.0 g/L)	Change (%)
GA_3_	9.94	12.57	**26.44**
IAA	16.27	21.62	**32.87**
Cytokinin	3.94	4.30	**9.13**
ABA	1.28	1.76	**37.83**
Total	29.32	38.94	**32.81**

### HPLC of phenolic and flavonoid compounds in wheat grains

The sum of total phenolic compounds (TPC) in wheat grains of control plants was 2887.5 µg/ g; biostimulant treatment accumulated phenolic compounds about 90% (Table 9). In the grains of control plants, the major group of phenolic compounds was flavonols which represent about 40% of TPC followed by hydroxybenzoic acid derivatives (HBAs, 14.3%); stilbene (resveratrol, 8.5%); hydroxycinammic acid derivatives (HCAs, 5.8%) and finally catechol (2%). Upon treating wheat plants with 1.0 g/L HLE, the pattern of phenolic compounds was quite different, the major group was flavonols (69.2%), followed by resveratrol (13.4%); naringenin (8.9%), HCAs (6.6%); and HBAs (1.7%); yet, small amount of ellagic acid was detected. Other phenolic compounds presented in Table 9 were detected in negligible amounts. It is clear from Table 9 that there was a decrease in the level of hydroxybenzoic acids and other compounds, conversely flavanols and resveratrol were accumulated which indicate their role as a storage phenolic compounds in the grains. Quercetin is abundant in the grains harvested from both control and treated plants. It recorded 1403 µg/ g which resemble 48.6% of TPC identified in the grains of control, it raised to 1.1 fold upon treatment. It is worth to mention that rutin and kaempferol were not detected in grains of control plants while, they appeared upon treatment. Myricetin represented 23% of TPC, upon treatment its level was decreased about 38%. Resveratrol (stilbene) was determined in the harvested grains of control plants (244.8 µg/ g), in response to treatment two fold increases was detected.

**Table 9:** HPLC analysis of phenolic and flavonoid compounds of wheat grains yielded from control and biostimulant (1.0 g/L HLE) treated plants, expressed as µg/g

No	Compound			Control	HLE (1.0 g/L)	Change (%)
1	Benzoic acid derivatives	Benzoic acid		346.645	325.156	-6
2		Gallic acid		39.013	4.063	-90
3		Syringic acid		25.826	2.688	-90
4		Vanillic acid		-	27.485	-
5	Cinnamic acid derivatives	Cinnamic acid		-	51.048	-
6		o- Coumaric acid		78.435	21.147	-73
7		p- Coumaric acid		-	3.062	-
8		Ferulic acid		27.964	3.909	-86
9		Caffeic acid		29.744	5.186	-83
10		Chlorogenic acid		30.976	5.722	-82
11	Flavonoids	Flavonols	Kaempferol	-	151.747	-
12			Quercetin	1403.844	2947.761	110
13			Myricetin	667.726	413.734	-38
14			Rutin	-	260.670	-
15		Naringenin (Flavanone)		-	483.538	-
16		Catechin (Flavanol)		2.702	4.018	49
17	Others	Resveratrol(stilbene)		244.800	731.846	199
18		Ellagic acid		-	11.996	-
19		Catechol		58.549	-	-
	Total			2887.467	5454.776	89

In this concern, several authors reported that wheat grains mainly contain ferulic, p-coumaric as well as other phenolic acids such as isoferulic, caffeic, o-coumaric, vanillic, sinapic, p-hydroxy-benzoic, chlorogenic and protocatechuric acids [86–88]. It was postulated that phenolic acids are the main antioxidant compounds in cereal grains; yet, in this investigation they appeared in small amounts except benzoic acid which gave the value of 347 µg/ grain (g), represents about 12% of TPC.

Benzoic acid may consider as essential precursor of primary and secondary metabolites. It can produce attractant compounds for pollinators; phytohormones, electron carriers and essential defense compounds with a pharmacological and medicinal properties [89]. Hernández et al., [90] analyzed the phenolic acids in the grains of 19 wheat cultivars among them *Triticum aestivum* recorded three HBAs and two HCAs. The predominant HCAs was ferulic acid (958 µg/g) followed by p-coumaric acid (21 µg/g) while, HBAs were syringic, p-hydroxybenzoic and vanillic acids (31.4, 13.4 and 4.53 µg/g, respectively). Suchowilska et al., [91] identified 11 phenolic acids in grains of wheat. The content of ferulic acid in Kamut® wheat grains (1455.8 µg /g) was almost 2.7 times higher in bread wheat (544.2 µg/g) and nearly two folds higher than in Polish wheat (734 µg/g). Polish wheat was characterized by the presence of p-coumaric, syringic, gallic and cinnamic acids (9.4, 41, 12 and 98 µg/g, respectively). Suchowilska et al., [91] reported that the flavonoid compounds in the grains of four wheat species such as quercetin, rutin,kaempferol, naringenin, and catechin exhibited insignificant variations among the studied cultivars. Suzuki and coworkers, [92] reported that rutin of Tartary buckwheat seeds plays an important role in antioxidant activity; during seed ripening, rutin level and rutinosidase activity. The rutinosidase activity in Tartary buckwheat seeds was sufficient to hydrolyze considerable rutin within few minutes to quercetin. The increased rutinosidase activity results in an increase in quercetin and rutinose levels and serves to supply quercetin as a peroxidase substrate [92].

## Conclusion

This study documented the influence of sequenced application of bioactive stimulant (henna) as a combination of seed priming and foliar spraying with 0.5, 1.0 and 5.0 g/L for two successive seasons. The results revealed the stimulatory effect of henna leaf extract (HLE) on the studied parameters of shoot and root systems up to 1.0 g/L dose, yet the highest dose (5.0 g/L) showed insignificant variations. Application of different concentrations of HLE elevated the level of photosynthetic pigments, sugars, phytohormones and soluble proteins with being 1.0 g/L the most effective dose. Additionally, application of 1.0 g/L markedly improved the quality of the yielded grains as revealed by increasing the content of soluble sugars, starch, gluten, soluble proteins and α-amylase activity by (23, 19, 50, 37 and 27%, respectively); the increments for phenolic compounds, flavonoids and tannins were (67, 87 and 23%, respectively), and for Ca, Na and Fe were (184, 10 and 10%, respectively). HPLC analysis of phytohormones, sugars and flavonoids exhibited significant increase in the grains yielded from plants treated with 1.0 g/ L HLE. Furthermore, wheat productivity recorded a striking increase in the grain yield (32%) and biological yield (35%) in response to plant treatment with 1.0 g/L HLE. Consequently, foliar application of 1.0 g/L was recommended for the farmers to improve quantity and quality of wheat.

## Data Availability

All data generated or analyzed during this study are included in this article and available from the corresponding author on reasonable request.
